# Sonic Hedgehog Signaling and Tooth Development

**DOI:** 10.3390/ijms21051587

**Published:** 2020-02-26

**Authors:** Akihiro Hosoya, Nazmus Shalehin, Hiroaki Takebe, Tsuyoshi Shimo, Kazuharu Irie

**Affiliations:** 1 Division of Histology, Department of Oral Growth and Development, School of Dentistry, Health Sciences University of Hokkaido, Ishikari-Tobetsu, Hokkaido 061-0293, Japan; shalehin@hoku-iryo-u.ac.jp (N.S.); takebeh@hoku-iryo-u.ac.jp (H.T.); irie@hoku-iryo-u.ac.jp (K.I.); 2 Division of Reconstructive Surgery for Oral and Maxillofacial Region, Department of Human Biology and Pathophysiology, School of Dentistry, Health Sciences University of Hokkaido, Ishikari-Tobetsu, Hokkaido 061-0293, Japan; shimotsu@hoku-iryo-u.ac.jp

**Keywords:** sonic hedgehog, tooth development, epithelial and mesenchymal interaction, Gli1, mesenchymal stem cell, lineage tracing analysis, stem cell marker

## Abstract

Sonic hedgehog (Shh) is a secreted protein with important roles in mammalian embryogenesis. During tooth development, Shh is primarily expressed in the dental epithelium, from initiation to the root formation stages. A number of studies have analyzed the function of Shh signaling at different stages of tooth development and have revealed that Shh signaling regulates the formation of various tooth components, including enamel, dentin, cementum, and other soft tissues. In addition, dental mesenchymal cells positive for Gli1, a downstream transcription factor of Shh signaling, have been found to have stem cell properties, including multipotency and the ability to self-renew. Indeed, Gli1-positive cells in mature teeth appear to contribute to the regeneration of dental pulp and periodontal tissues. In this review, we provide an overview of recent advances related to the role of Shh signaling in tooth development, as well as the contribution of this pathway to tooth homeostasis and regeneration.

## 1. Introduction

Hedgehog (Hh) signaling has been reported to have important roles in the development of many organs including craniofacial tissues such as palate, lip, salivary gland [1,2,3,4,5], as well as tooth [6]. This signaling requires primary cilia that function in intraflagellar transport (IFT) [7]. Disruption of IFT trafficking from the base to the tip of the cilium in *kif3a*-deficient mice results in phenotypes similar to the loss of Hh signaling, such as tooth dysplasia [8]. Under quiescent conditions, when Hh signaling is not activated, Patched (Ptch), a receptor of three hedgehog orthologs, including Sonic hedgehog (Shh), Indian hedgehog, and Desert hedgehog, represses Smoothened (Smo). Canonical Hh signaling is mediated via Smo activation. When the hedgehog ligand binds Ptch, it relieves this suppression and Smo accumulates in the tip of the primary cilium. Accordingly, Gli becomes dissociated from Suppressor of Fused (Sufu), a negative regulator of the Shh signaling. It then leads to the activation of Gli transcription factors and the downstream hedgehog signaling pathway [9]. Gli transcription factors have DNA-binding zinc finger domains that bind to sequences on their target genes to initiate or inhibit their transcription [10]. In contrast, non-canonical Hh signaling occurs through Patched1, independently of Smo and Gli [11].

**Figure 1:**
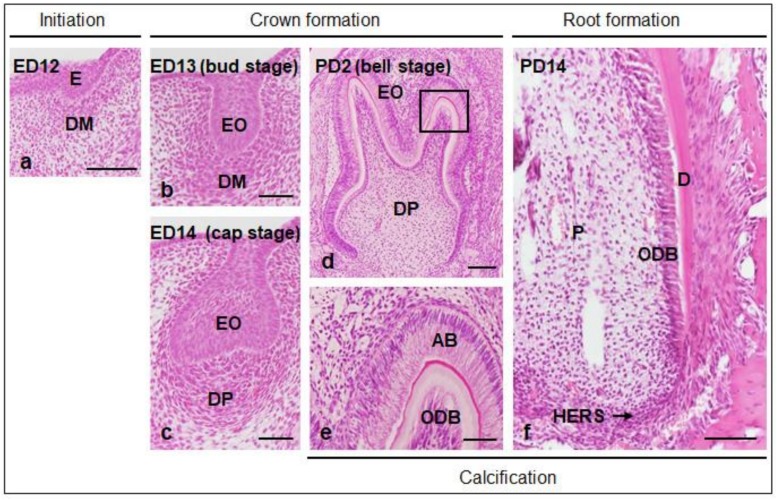
Process of molar tooth development in mouse. (**a–e**) Tooth development begins with thickening of the oral epithelium (E) and progresses to crown (bud, cap, and bell stages) and root formation stages. Calcification of enamel, dentin (D), and cementum occurs after the bell stage. The formation stages “initiation,” “crown formation,” and “root formation” correspond to the terms in Table 1. Higher magnification of the boxed region in “d” is shown in “e.” AB, ameloblast; DM, dental mesenchyme; DP, dental papillae; ED, embryonic day; EO, enamel organ; HERS, Hertwig’s epithelial root sheath; ODB, odontoblast; P, pulp; PD, postnatal day. Scale bars = 100 μm (**a**), 50 μm (**b**–**d**,**f**), 25 μm (**e**).

Tooth germ is composed of both epithelial and mesenchymal tissues, with dental epithelial tissue originating from the oral epithelium. However, unlike the nearby oral epithelium, the dental epithelium expresses *Shh* [12,13,14,15,16]. During the period of tooth crown formation, Shh-expressing cells are strictly localized in the dental epithelium, including the enamel knot that corresponds to future cusps, as well as ameloblast-lineage cells [17,18,19]. On the other hand, Ptch-positive cells and its downstream proteins are located in the dental mesenchyme in the absence of *Shh* expression [20]. Therefore, it is believed that an epithelial-mesenchymal interaction exists in which *Shh* expressed in the epithelium acts on Ptch-positive mesenchymal cells during tooth development. Conversely, several reports have demonstrated that cells in the dental mesenchyme regulate Shh expression in the dental epithelium [21,22,23,24,25,26,27,28,29,30]. It has been shown that expression levels of *Shh* in the dental epithelium are decreased in *runt-related transcription factor 2* (*Runx2*) mutant mice [31]. Runx2 is an essential transcription factor for osteoblast differentiation and is expressed in both osteogenic- and odontogenic-lineage cells, indicating that dental mesenchymal cells may regulate Shh expression in the epithelium. Furthermore, it has been reported that Shh signaling is strictly regulated in certain types of cells and is required for cellular proliferation and differentiation during different stages of tooth development (Table 1, Figure 1).

In this review, we focus on the functions of Shh signaling related to tooth development. In addition, we introduce recent findings concerning the relationship between Shh signaling and stem cell maintenance, with an emphasis on the potential of Shh signaling for the regeneration of dental tissues.

## 2. Shh Is Important for Epithelial Invagination at the Initiation of Tooth Development

Tooth development is regulated by reciprocal interactions occurring between epithelial and mesenchymal tissues. These interactions are controlled by several conserved signaling molecules, including bone morphogenetic proteins (BMPs), fibroblast growth factor, Wnts, and Shh [24,25]. At the beginning of tooth development, the oral epithelium actively grows and invaginates toward the mesenchyme. The expression of *Shh* has been demonstrated in the thickening epithelium at the site where tooth formation will occur [31,45,46,47]. Shh signaling related molecules such as *Ptch*, *Smo*, *Gli1*, *Gli2*, and *Gli3* are also expressed in the dental mesenchyme around the thickening epithelium [32,48]. The implantation of Shh-soaked beads into the dental mesenchyme has been shown to enhance the expression of *Ptch* and *Gli1* at this site, subsequently resulting in an irregular shape of the thickening epithelium. On the other hand, the implantation of Shh-soaked beads into the oral epithelium, but not around the thickening dental epithelium, induces an ectopic epithelial invagination [33]. Enhancement of *Shh* expression in the dental epithelium using the Keratin 14 promoter inhibits cellular proliferation and arrests tooth development during the early stage [34]. Conversely, the inhibition of Shh signaling by cyclopamine, an antagonist of Smo, inhibits the invagination and extension of the oral epithelium into the dental mesenchyme [33]. In *Gli2* and *Gli3* double-mutant mice, although epithelial thickening is observed in the oral epithelium, the epithelium does not proceed to form the enamel organ [32]. Therefore, Shh signaling appears to have an important role in dental epithelial cellular proliferation and invagination.

## 3. Shh Regulates Enamel Formation

During tooth development, the invaginated dental epithelium extends and forms the enamel organ. Epithelial cells in this tissue can be divided into three types of tissues, namely, the inner and outer epithelia and the stellate reticulum. During tooth crown formation, the cells in the inner enamel epithelium differentiate into ameloblasts that form the enamel. The inner enamel epithelium at this stage expresses both *Shh* and *Ptch* [24,49,50,51,52,53,54], and suppression of these expressions results in the inhibition of the proliferative activity of the epithelial cells [35]. In addition, it has been demonstrated that inhibition of Shh signaling in tooth germ using a neutralizing antibody suppresses ameloblast differentiation [36].

*Shh* is also expressed in enamel-secreting ameloblasts [55,56,57]. As such, the loss of Shh signaling in ameloblast-lineage cells using genetic modification techniques has been shown to cause unpolarized ameloblast differentiation and enamel hypoplasia, resulting in the disruption of normal tooth morphology [37]. Therefore, Shh signaling appears to have multiple roles, which include the proliferation and differentiation of cells in the inner enamel epithelium and in differentiated ameloblasts.

## 4. Shh Signaling Functions in the Dental Mesenchyme and Is Involved in Tooth Morphogenesis

It has been reported that the inactivation of Shh signaling in the dental epithelium results in the formation of small teeth with the disappearance of Ptch1- and Gli1-positive cells in the dental mesenchyme [39]. Suppression of Sufu in dental mesenchymal cells results in deletion of primary enamel knot in the enamel organ as well as retardation of transition from bud to cap stage of tooth development [40]. It has also been demonstrated that crown size depends on the contact area between the *Shh*-expressing inner enamel epithelium and the dental mesenchyme [54]. These findings indicate that Shh signaling may regulate cellular proliferation in the dental mesenchyme, thereby controlling tooth morphogenesis [38,41,58].

## 5. Deletion of Shh Signaling in Hertwig’s Epithelial Root Sheath (HERS) Suppresses Tooth Root Elongation

After crown formation, the inner and outer enamel epithelium fuse at the lower edge of the enamel organ, forming a bilayered tissue referred to as HERS. Morphologically, the HERS bends inward during the early stages of root formation and grows between the dental papilla and dental follicle. In general, the HERS has been accepted as the principal structure controlling root formation, as this tissue disappears upon completion of root formation. Recent studies have demonstrated that growth factors, including BMPs and transforming growth factor-beta, mediate reciprocal epithelial-mesenchymal interactions during tooth root development [21,45,46,59]. It has also been shown that the epithelial cells of the HERS secrete Shh [42,43,60]. In this process, via Shh signaling, dental mesenchymal cells expressing Ptch are stimulated to form the root dentin [35,36].

*Nuclear factor Ic* (*Nfic*) knockout mice have normal tooth crowns, but a defect of tooth root formation can be observed in the molars [61]. This suggests that *Nfic* has an essential role in tooth root formation. The loss of Shh in the HERS has been shown to inhibit the expression of *Nfic* in the dental mesenchyme around the HERS [43]. Therefore, it is considered that Shh is an important signaling molecule of the epithelial-mesenchymal interaction and regulates tooth root formation.

## 6. Signaling Pathways of BMP-SHH and SHH-BMP Regulate Tooth Root Formation

While evidence suggests that Shh signaling has an important role for tooth root development [42,44], the mechanisms of this process remain controversial. In the process of tooth root development, as mentioned above, BMPs are important signaling molecules that regulate epithelial-mesenchymal tissue interactions [45,46,62]. In particular, BMPs principally function via receptor complexes consisting of BMP receptor types I (BMPR-I) and II (BMPR-II) [63]. BMPs activate these receptors upon binding, which then leads to the phosphorylation of R-Smads. Phosphorylated R-Smads subsequently interacts with Smad4 to form a complex, which is translocated to the nucleus [64,65]. This complex then induces the expression of downstream proteins, including Runx2, which are essential transcription factors for hard tissue-forming cell differentiation [66].

The inactivation of Smad4 in the dental epithelium using Keratin 14-Cre; Smad4fl/fl mice have been shown to cause the absence of *Shh* expression in the HERS, resulting in the formation of short tooth roots [43]. In addition, a similar phenotype is observed in mice with mutated BMPR-I in the dental epithelium [67]. In the dental mesenchyme, some positive cells for downstream proteins of Shh signaling are known to be present, including Gli1. The inhibition of BMP signaling in these Gli1-positive cells results in a failure of root dentin formation [43,67]. Therefore, it can be speculated that certain key molecules regulated by Shh signaling may be closely associated with tooth root development, suggesting that BMP and Shh signaling pathways may be regulators of tooth root formation.

## 7. Gli1-Expressing Cells Possess Stem Cell Properties in Mature Tooth

**Figure 2:**
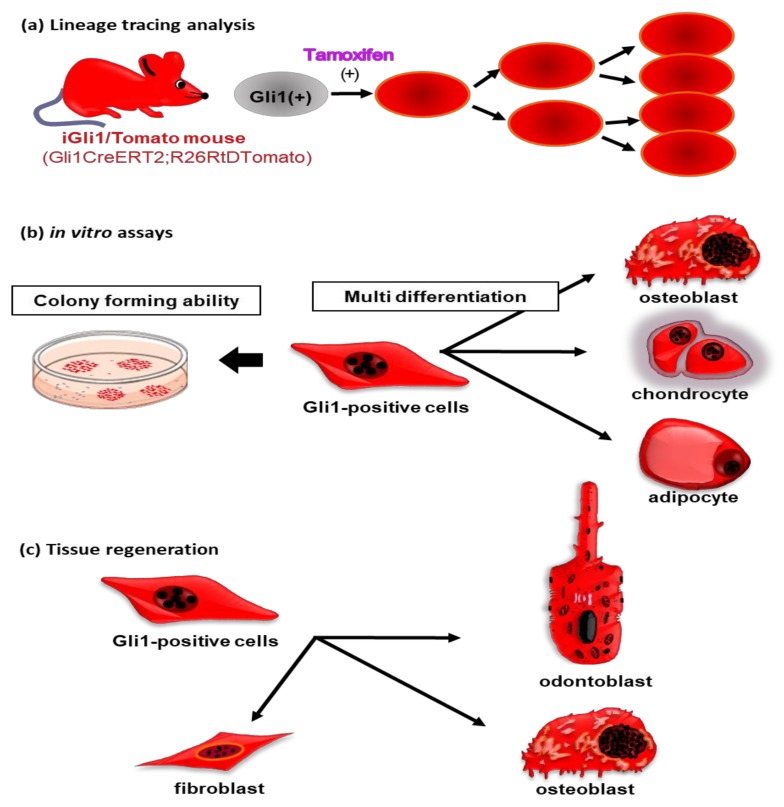
Schematic illustrations of characteristics of Gli1-positive cells in mature teeth. (**a**) After tamoxifen administration in iGli1/Tomato mice, Gli1-positive cells are shown to express Tomato red fluorescence. Cells that once expressed Tomato red fluorescence continuously emit this fluorescence even after cell division. Using this system, it is possible to trace the differentiation process of Gli1-positive cells and their progeny cells. (**b**) Gli1-positive cells exhibit high colony-forming unit fibroblast (CFU-F) activity. These cells also have trilineage potential to form osteoblasts, chondrocytes, and adipocytes in vitro. (**c**) After tooth transplantation into subcutaneous tissue, Gli1-positive cells differentiate into odontoblasts, osteoblasts, and fibroblasts during tissue regeneration.

Multipotent mesenchymal stem cells have been described in a variety of tissues with varying developmental origins and physiological functions [68,69]. Although human permanent and deciduous teeth are known to contain mesenchymal stem cells in the periodontal ligament and dental pulp [70,71,72], visualization of these cells has yet to be achieved. Recently, iGli1/Tomato mice, which are transgenic for the *Gli1CreERT2; R26RtDTomato* gene [73,74], have been used for lineage tracing analysis of Gli1-positive cells in various organs [75,76,77,78,79,80,81]. In this mouse model, Gli1-positive cells were shown to express the Cre recombinase-mutated estrogen receptor (CreERT2). Since CreERT2 is only active in the presence of tamoxifen, Gli1-positive cells start to express Tomato red fluorescence after tamoxifen administration. Tomato red fluorescence is also observed in the daughter cells of Gli1-positive cells after cell division. Therefore, this system can be used to continuously trace Gli1-positive cells and their daughter cells (Figure 2a).

**Figure 3:**
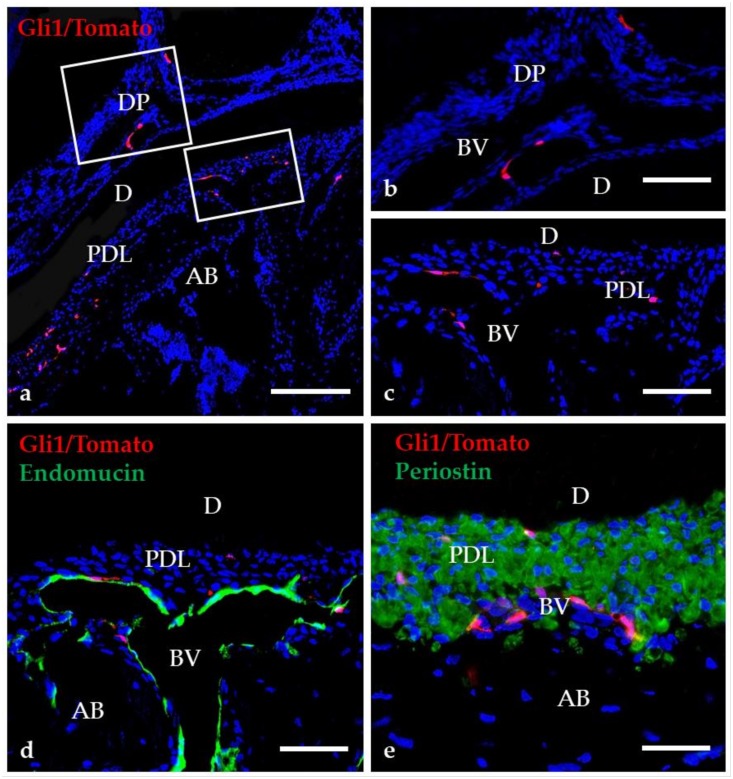
Distribution of Gli1-positive cells in mature teeth. Higher magnification of the boxed region in “a” are shown in “b”–“e.” (**a–c**) Gli1-positive cells are present in the dental pulp (DP) and the periodontal ligament (PDL). (**d–e**) The merged image of Endomucin and Periostin with Gli1/Tomato fluorescence demonstrate that most Gli1/Tomato-positive cells are distributed near blood vessels (BV). AB, alveolar bone; D, dentin. Scale bars = 100 μm (**a**), 25 μm (**b–e**).

In a previous study, we revealed that Gli1-positive cells are present in the dental pulp and the periodontal ligament in mature teeth [82]. These cells are barely detected around the blood vessels in mature tooth (Figure 3a–e). In addition, Gli1-positive cells have been identified as mesenchymal stem cells with the ability to self-renew and with trilineage differentiation potential (Figure 2b). Although Gli1-positive cells are quiescent under normal conditions after the completion of tooth formation, they can proliferate after tissue injury, contributing to tissue repair (Figure 2c). In the following chapters, recent studies demonstrating the stem cell abilities of Gli1-positive cells during tooth development will be discussed.

## 8. Gli1-Positive Cells Supply Ameloblast-Lineage Cells in the Rodent Incisor

Since rodent incisors erupt continuously throughout the life of the animal, epithelial stem cells that differentiate into enamel-forming ameloblasts are present in the dental epithelium at the posterior apex of the incisor [83]. In addition, Gli1-positive cells are distributed in proximity to *Shh*-expressing cells in the cervical loop of the incisor. These cells have been shown to be co-localized with bromodeoxyuridine label-retaining cells, suggesting the presence of both stem cells and transit-amplifying cells [84]. Using lineage tracing analysis, Gli1-positive cells in the dental epithelium of the mouse incisor have been shown to proliferate and differentiate into ameloblasts [85]. Furthermore, since the formation of enamel can be blocked in the mouse incisor by the administration of hedgehog pathway inhibitors [36,37,85], Shh signaling may contribute to both the maintenance of epithelial stem cells and ameloblast differentiation.

## 9. Gli1-Positive Cells Are Mesenchymal Stem Cells in Developing Tooth

Mesenchymal cells in tooth germ have been considered to originate from the cranial neural crest [86]. In the mouse incisor, it has been reported that most cells originating from the cranial neural crest express *Gli1* and are localized at the posterior apex of the dental mesenchyme without a high proliferation ability. These cells expand and populate the entire dental pulp, as well as the periodontal ligament [87,88]. Zhao et al. [87] suggested that Shh secreted by sensory nerves, not the dental epithelium, is important for the maintenance of these Gli1-positive cells in the dental mesenchyme of the mouse incisor. Similarly, it has been reported that the nerve-derived Shh is involved in supporting the stem cell niche in hair follicle for its development and regeneration [89].

Just after the beginning of root formation stage of the mouse molar, the HERS secrets Shh [42,43]. Gli1-positive cells are then distributed in the dental mesenchyme around the HERS [44,67,90] and proliferate as the tooth root elongates, differentiating into root-forming cells such as odontoblasts, cementoblasts, and fibroblasts in the dental pulp and the periodontal ligament [44,91]. These Gli1-positive cells have also been shown to have multilineage potential and high colony-forming unit fibroblast (CFU-F) activity *in vitro* [90]. Furthermore, root elongation is not observed in tooth germ lacking Gli1-positive cells during the root formation stage [44]. Therefore, Gli1-positive cells are believed to supply the cells involved in tooth root formation. These results also indicate that Gli1 may be a useful marker of mesenchymal stem cells in the developing tooth (Table 2).

## 10. Can Shh Signaling Be a Target for Tooth Regeneration Therapy?

Cell replacement therapies using undifferentiated cells are considered to be one of the most effective methods for cellular and tissue regeneration. As such, regenerative therapies using stem cells have been widely studied in a variety of organs [92,93]. This approach is considered to be constructive as it promotes healing in the original cells. In vivo studies have shown that, after tooth transplantation into the subcutaneous tissue, stem and undifferentiated cells can differentiate into odontoblasts [94,95], cementoblasts, and osteoblasts [96,97]. Interestingly, the majority of cells with this regenerative ability have been shown to express *Gli1* (Figure 2c).The collection of dental pulp and periodontal ligament cells containing Gli1-positive cells from teeth extracted for orthodontic reasons or from nonfunctional third molars is possible. In addition, the elucidation of mechanisms concerning stem and undifferentiated cell maintenance by Shh signaling may lead to the application of Gli1-positive cells for tooth regeneration. However, in practical terms, a large number of replacement cells would be required because stem cells in tooth are present only in a limited number. Furthermore, in vitro culture systems to expand these stem cells, while maintaining their unique characteristics, have not been established. Therefore, a better understanding of the mechanisms underlying the maintenance of stemness, as well as tooth cell differentiation in Gli1-positive cells, may lead to more effective biologically activating therapies than are currently offered by traditional dental treatments.

## 11. Conclusions

Shh signaling is deeply involved in tooth formation and has different functions at each stage of tooth development. Therefore, a greater understanding of tooth formation may accelerate the development of novel regenerative and restorative therapies. Indeed, recent studies have shown that cells expressing *Gli1*, a downstream factor of Shh signaling, are mesenchymal cells in both developing and mature teeth. Thus, it is expected that additional functions of Shh signaling in tooth formation, as well as the regulatory mechanism of stem cell properties in the dental mesenchyme, will be elucidated and lead to the development of new dental therapies.

## Figures and Tables

**Table 1:** Roles of Shh signaling during tooth development.

Stage	Expressing Cells		Function	References
	*Shh*	*Ptch, Smo, Gli*		
Initiation	Epithelium	Dental mesenchyme	Epithelial invagination	[32,33,34]
Crown formation	Enamel organ	Enamel organ	Ameloblast differentiation	[35,36,37,38]
Calcification	Enamel organ	Dental papillae	Tooth morphogenesis	[37,38,39,40,41]
Root formation	HERS	Dental mesenchyme	Root elongation	[35,42,43,44]

**Table 2:** Differentiatial ability of Gli1-positive cells in mouse developing tooth.

Tooth	Localization of Gli1-Positive Cells	Differentiating Cells	References
Incisor	Epithelium in cervical loop	Ameloblasts	[85]
	Mesenchyme around cervical loop	Crown forming cells without ameloblasts	[87,88]
Molar	Mesenchyme around HERS	Root forming cells	[44,67,90]

## Abbreviations

BMP	Bone Morphogenetic Protein
BMPR	Bone Morphogenetic Protein Receptor
CreERT2	Cre Recombinase-mutated Estrogen Receptor
HERS	Hertwig’s Epithelial Root Sheath
Hh	Hedgehog
IFT	Intraflagellar Transport
Ptch	Patched
Runx2	Runt-related Transcription Factor 2
Shh	Sonic Hedgehog
Smo	Smoothend

## References

[1] Kurosaka H. (2015). The roles of hedgehog signaling in upper lip formation. Biomed. Res. Int..

[2] Xavier G.M., Seppala M., Barrell W., Birjandi A.A., Geoghegan F., Cobourne M.T. (2016). Hedgehog receptor function during craniofacial development. Dev. Biol..

[3] Dworkin S., Boglev Y., Owens H., Goldie S.J. (2016). The role of Sonic hedgehog in craniofacial patterning, morphogenesis and cranial neural crest survival. J. Dev. Biol..

[4] Elliott K.H., Millington G., Brugmann S.A. (2018). A novel role for cilia-dependent Sonic hedgehog signaling during submandibular gland development. Dev. Dyn..

[5] Abramyan J. (2019). Hedgehog signaling and embryonic craniofacial disorders. J. Dev. Biol..

[6] Seppala M., Fraser G.J., Birjandi A.A., Xavier G.M., Cobourne M.T. (2017). Sonic hedgehog signaling and development of the dentition. J. Dev. Biol..

[7] Goetz S.C., Anderson K.V. (2010). The Primary Cilium: A signaling center during vertebrate development. Nat. Rev. Genet..

[8] Liu B., Chen S., Cheng D., Jing W., Helms J.A. (2014). Primary cilia integrate hedgehog and Wnt signaling during tooth development. J. Dent. Res..

[9] Briscoe J., Therond P.P. (2013). The mechanisms of hedgehog signaling and its roles in development and disease. Nat. Rev. Mol. Cell Biol..

[10] Sasaki H., Hui C., Nakafuku M., Kondoh H. (1997). A binding site for Gli proteins is essential for HNF-3beta floor plate enhancer activity in transgenics and can respond to Shh in vitro. Development.

[11] Yang J., Andre P., Ye L., Yang Y.Z. (2015). The hedgehog signaling pathway in bone formation. Int. J. Oral Sci..

[12] Jernvall J., Keränen S.V., Thesleff I. (2000). Evolutionary modification of development in mammalian teeth: Quantifying gene expression patterns and topography. Proc. Natl. Acad. Sci. USA.

[13] Koyama E., Wu C., Shimo T., Iwamoto M., Ohmori T., Kurisu K., Ookura T., Bashir M.M., Abrams W.R., Tucker T. (2001). Development of stratum intermedium and its role as a Sonic hedgehog-signaling structure during odontogenesis. Dev. Dyn..

[14] Miyado M., Ogi H., Yamada G., Kitoh J., Jogahara T., Oda S., Sato I., Miyado K., Sunohara M. (2007). Sonic hedgehog expression during early tooth development in suncus murinus. Biochem. Biophys. Res. Commun..

[15] Hovorakova M., Smrckova L., Lesot H., Lochovska K., Peterka M., Peterkova R. (2013). Sequential Shh expression in the development of the mouse upper functional incisor. J. Exp. Zool. B Mol. Dev. Evol..

[16] Hu X., Zhang S., Chen G., Lin C., Huang Z., Chen Y., Zhang Y. (2013). Expression of SHH signaling molecules in the developing human primary dentition. BMC Dev. Biol..

[17] Yamanaka A., Yasui K., Sonomura T., Iwai H., Uemura M. (2010). Development of deciduous and permanent dentitions in the upper jaw of the house shrew (Suncus murinus). Arch. Oral Biol..

[18] Hovorakova M., Prochazka J., Lesot H., Smrckova L., Churava S., Boran T., Kozmik Z., Klein O., Peterkova R., Peterka M. (2011). Shh expression in a rudimentary tooth offers new insights into development of the mouse incisor. J. Exp. Zool. B Mol. Dev. Evol..

[19] Nakatomi C., Nakatomi M., Saito K., Harada H., Ohshima H. (2015). The enamel knot-like structure is eternally maintained in the apical bud of postnatal mouse incisors. Arch. Oral Biol..

[20] Khan M., Seppala M., Zoupa M., Cobourne M.T. (2007). Hedgehog pathway gene expression during early development of the molar tooth root in the mouse. Gene Expr. Patterns.

[21] Zhang Y., Zhang Z., Zhao X., Yu X., Hu Y., Geronimo B., Fromm S.H., Chen Y.P. (2000). A new function of BMP4: Dual role for BMP4 in regulation of Sonic hedgehog expression in the mouse tooth germ. Development.

[22] Sarkar L., Cobourne M., Naylor S., Smalley M., Dale T., Sharpe P.T. (2000). Wnt/Shh interactions regulate ectodermal boundary formation during mammalian tooth development. Proc. Natl. Acad. Sci. USA.

[23] Ten Berge D., Brouwer A., Korving J., Reijnen M.J., van Raaij E.J., Verbeek F., Gaffield W., Meijlink F. (2001). Prx1 and Prx2 are upstream regulators of sonic hedgehog and control cell proliferation during mandibular arch morphogenesis. Development.

[24] Handrigan G.R., Richman J.M. (2010). A network of Wnt, hedgehog and BMP signaling pathways regulates tooth replacement in snakes. Dev. Biol..

[25] Ahn Y., Sanderson B.W., Klein O.D., Krumlauf R. (2010). Inhibition of Wnt signaling by Wise (Sostdc1) and negative feedback from Shh controls tooth number and patterning. Development.

[26] Cho S.W., Kwak S., Woolley T.E., Lee M.J., Kim E.J., Baker R.E., Kim H.J., Shin J.S., Tickle C., Maini P.K. (2011). Interactions between Shh, Sostdc1 and Wnt signaling and a new feedback loop for spatial patterning of the teeth. Development.

[27] Tokita M., Chaeychomsri W., Siruntawineti J. (2013). Developmental basis of toothlessness in turtles: Insight into convergent evolution of vertebrate morphology. Evolution.

[28] Aurrekoetxea M., Irastorza I., García-Gallastegui P., Jiménez-Rojo L., Nakamura T., Yamada Y., Ibarretxe G., Unda F.J. (2016). Wnt/β-catenin regulates the activity of epiprofin/Sp6, SHH, FGF, and BMP to coordinate the stages of odontogenesis. Front. Cell Dev. Biol..

[29] Sagai T., Amano T., Maeno A., Kiyonari H., Seo H., Cho S.W., Shiroishi T. (2017). SHH signaling directed by two oral epithelium-specific enhancers controls tooth and oral development. Sci. Rep..

[30] Seo H., Amano T., Seki R., Sagai T., Kim J., Cho S.W., Shiroishi T. (2018). Upstream enhancer elements of Shh regulate oral and dental patterning. J. Dent. Res..

[31] Wang X.P., Aberg T., James M.J., Levanon D., Groner Y., Thesleff I. (2005). Runx2 (Cbfa1) inhibits Shh signaling in the lower but not upper molars of mouse embryos and prevents the budding of putative successional teeth. J. Dent. Res..

[32] Hardcastle Z., Mo R., Hui C.C., Sharpe P.T. (1998). The Shh signalling pathway in tooth development: Defects in Gli2 and Gli3 mutants. Development.

[33] Li J., Chatzeli L., Panousopoulou E., Tucker A.S., Green J.B. (2016). Epithelial stratification and placode invagination are separable functions in early morphogenesis of the molar tooth. Development.

[34] Cobourne M.T., Xavier G.M., Depew M., Hagan L., Sealby J., Webster Z., Sharpe P.T. (2009). Sonic hedgehog signalling inhibits palatogenesis and arrests tooth development in a mouse model of the nevoid basal cell carcinoma syndrome. Dev. Biol..

[35] Wu C., Shimo T., Liu M., Pacifici M., Koyama E. (2003). Sonic hedgehog functions as a mitogen during bell stage of odontogenesis. Connect. Tissue Res..

[36] Koyama E., Wu C., Shimo T., Pacifici M. (2003). Chick Limbs With Mouse Teeth: An effective in vivo culture system for tooth germ development and analysis. Dev. Dyn..

[37] Gritli-Linde A., Bei M., Maas R., Zhang X.M., Linde A., McMahon A.P. (2002). Shh signaling within the dental epithelium is necessary for cell proliferation, growth and polarization. Development.

[38] Yu J.C., Fox Z.D., Crimp J.L., Littleford H.E., Jowdry A.L., Jackman W.R. (2015). Hedgehog signaling regulates dental papilla formation and tooth size during zebrafish odontogenesis. Dev. Dyn..

[39] Dassule H.R., Lewis P., Bei M., Maas R., McMahon A.P. (2000). Sonic hedgehog regulates growth and morphogenesis of the tooth. Development.

[40] Li J., Xu J., Cui Y., Wang L., Wang B., Wang Q., Zhang X., Qiu M., Zhang Z. (2019). Mesenchymal Sufu regulates development of mandibular molars via Shh signaling. J. Dent. Res..

[41] Ishida K., Murofushi M., Nakao K., Morita R., Ogawa M., Tsuji T. (2011). The regulation of tooth morphogenesis is associated with epithelial cell proliferation and the expression of Sonic hedgehog through epithelial-mesenchymal interactions. Biochem. Biophys. Res. Commun..

[42] Nakatomi M., Morita I., Eto K., Ota M.S. (2006). Sonic hedgehog signaling is important in tooth root development. J. Dent. Res..

[43] Huang X., Xu X., Bringas P., Hung Y.P., Chai Y. (2010). Smad4-Shh-Nfic signaling cascade-mediated epithelial-mesenchymal interaction is crucial in regulating tooth root development. J. Bone Miner. Res..

[44] Liu Y., Feng J., Li J., Zhao H., Ho T.V., Chai Y. (2015). An Nfic-hedgehog signaling cascade regulates tooth root development. Development.

[45] Thesleff I. (2003). Epithelial-mesenchymal signalling regulating tooth morphogenesis. J. Cell Sci..

[46] Tucker A., Sharpe P. (2004). The cutting-edge of mammalian development; how the embryo makes teeth. Nat. Rev. Genet..

[47] Buchtová M., Handrigan G.R., Tucker A.S., Lozanoff S., Town L., Fu K., Diewert V.M., Wicking C., Richman J.M. (2008). Initiation and patterning of the snake dentition are dependent on Sonic hedgehog signaling. Dev. Biol..

[48] Cobourne M.T., Miletich I., Sharpe P.T. (2004). Restriction of sonic hedgehog signalling during early tooth development. Development.

[49] Koyama E., Yamaai T., Iseki S., Ohuchi H., Nohno T., Yoshioka H., Hayashi Y., Leatherman J.L., Golden E.B., Noji S. (1996). Polarizing activity, Sonic hedgehog, and tooth development in embryonic and postnatal mouse. Dev. Dyn..

[50] Peterková R., Peterka M., Viriot L., Lesot H. (2000). Dentition development and budding morphogenesis. J. Craniofac. Genet. Dev. Biol..

[51] Kriangkrai R., Iseki S., Eto K., Chareonvit S. (2006). Dual odontogenic origins develop at the early stage of rat maxillary incisor development. Anat. Embryol. Berl..

[52] Nunes F.D., Valenzuela Mda G., Rodini C.O., Massironi S.M., Ko G.M. (2007). Localization of Bmp-4, Shh and Wnt-5a transcripts during early mice tooth development by in situ hybridization. Braz. Oral Res..

[53] Zhang L., Hua F., Yuan G.H., Zhang Y.D., Chen Z. (2008). Sonic hedgehog signaling is critical for cytodifferentiation and cusp formation in developing mouse molars. J. Mol. Histol..

[54] Handrigan G.R., Richman J.M. (2010). Autocrine and paracrine Shh signaling are necessary for tooth morphogenesis, but not tooth replacement in snakes and lizards (Squamata). Dev. Biol..

[55] Iseki S., Araga A., Ohuchi H., Nohno T., Yoshioka H., Hayashi F., Noji S. (1996). Sonic hedgehog is expressed in epithelial cells during development of whisker, hair, and tooth. Biochem. Biophys. Res. Commun..

[56] Kumamoto H., Ohki K., Ooya K. (2004). Expression of Sonic hedgehog (SHH) signaling molecules in ameloblastomas. J. Oral Pathol. Med..

[57] Takahashi S., Kawashima N., Sakamoto K., Nakata A., Kameda T., Sugiyama T., Katsube K., Suda H. (2007). Differentiation of an ameloblast-lineage cell line (ALC) is induced by Sonic hedgehog signaling. Biochem. Biophys. Res. Commun..

[58] Jackman W.R., Yoo J.J., Stock D.W. (2010). Hedgehog signaling is required at multiple stages of zebrafish tooth development. BMC Dev. Biol..

[59] Thesleff I., Sharpe P. (1997). Signalling networks regulating dental development. Mech Dev.

[60] Bae W.J., Auh Q.S., Lim H.C., Kim G.T., Kim H.S., Kim E.C. (2016). Sonic hedgehog promotes cementoblastic differentiation via activating the BMP pathways. Calcif. Tissue Int..

[61] Steele-Perkins G., Butz K.G., Lyons G.E., Zeichner-David M., Kim H.J., Cho M.I., Gronostajski R.M. (2003). Essential role for NFI-C/CTF transcription-replication factor in tooth root development. Mol. Cell Biol..

[62] Nie X., Luukko K., Kettunen P. (2006). BMP signalling in craniofacial development. Int. J. Dev. Biol..

[63] Derynck R., Zhang Y.E. (2003). Smad-dependent and Smad-independent pathways in TGF-beta family signalling. Nature.

[64] Derynck R., Zhang Y., Feng X.H. (1998). Smads: Transcriptional activators of TGF-beta responses. Cell.

[65] Shi Y., Massague J. (2003). Mechanisms of TGF-beta signaling from cell membrane to the nucleus. Cell.

[66] Komori T., Yagi H., Nomura S., Yamaguchi A., Sasaki K., Deguchi K., Shimizu Y., Bronson R.T., Gao Y.H., Inada M. (1997). Targeted disruption of Cbfa1 results in a complete lack of bone formation owing to maturational arrest of osteoblasts. Cell.

[67] Li J., Feng J., Liu Y., Ho T.V., Grimes W., Ho H.A., Park S., Wang S., Chai Y. (2015). BMP-SHH signaling network controls epithelial stem cell fate via regulation of its niche in the developing tooth. Dev. Cell.

[68] Friedenstein A.J., Petrakova K.V., Kurolesova A.I., Frolova G.P. (1968). Heterotopic of bone marrow. Analysis of precursor cells for osteogenic and hematopoietic tissues. Transplantation.

[69] Bianco P., Robey P.G., Simmons P.J. (2008). Mesenchymal stem cells: Revisiting history, concepts, and assays. Cell Stem Cell.

[70] Gronthos S., Mankani M., Brahim J., Robey P.G., Shi S. (2000). Postnatal human dental pulp stem cells (DPSCs) in vitro and in vivo. Proc. Natl. Acad. Sci. USA.

[71] Miura M., Gronthos S., Zhao M., Lu B., Fisher L.W., Robey P.G., Shi S. (2003). SHED: Stem cells from human exfoliated deciduous teeth. Proc. Natl. Acad. Sci. USA.

[72] Seo B.M., Miura M., Gronthos S., Bartold P.M., Batouli S., Brahim J., Young M., Robey P.G., Wang C.Y., Shi S. (2004). Investigation of multipotent postnatal stem cells from human periodontal ligament. Lancet.

[73] Ahn S., Joyner A.L. (2004). Dynamic changes in the response of cells to positive hedgehog signaling during mouse limb patterning. Cell.

[74] Madisen L., Zwingman T.A., Sunkin S.M., Oh S.W., Zariwala H.A., Gu H., Ng L.L., Palmiter R.D., Hawrylycz M.J., Jones A.R. (2010). A robust and high-throughput Cre reporting and characterization system for the whole mouse brain. Nat. Neurosci..

[75] Kramann R., Schneider R.K., DiRocco D.P., Machado F., Fleig S., Bondzie P.A., Henderson J.M., Ebert B.L., Humphreys B.D. (2015). Perivascular Gli1+ progenitors are key contributors to injury-induced organ fibrosis. Cell Stem Cell.

[76] Zhao H., Feng J., Ho T.V., Grimes W., Urata M., Chai Y. (2015). The suture provides a niche for mesenchymal stem cells of craniofacial bones. Nat. Cell. Biol..

[77] Liu C., Rodriguez K., Yao H.H. (2016). Mapping lineage progression of somatic progenitor cells in the mouse fetal testis. Development.

[78] Kramann R., Goettsch C., Wongboonsin J., Iwata H., Schneider R.K., Kuppe C., Kaesler N., Chang-Panesso M., Machado F.G., Gratwohl S. (2016). Adventitial MSC-like cells are progenitors of vascular smooth muscle cells and drive vascular calcification in chronic kidney disease. Cell Stem Cell.

[79] Shi Y., He G., Lee W.C., McKenzie J.A., Silva M.J., Long F. (2017). Gli1 identifies osteogenic progenitors for bone formation and fracture repair. Nat. Commun..

[80] Degirmenci B., Valenta T., Dimitrieva S., Hausmann G., Basler K. (2018). GLI1-expressing mesenchymal cells form the essential Wnt-secreting niche for colon stem cells. Nature.

[81] Ó hAinmhire E., Wu H., Muto Y., Donnelly E.L., Machado F.G., Fan L.X., Chang-Panesso M., Humphreys B.D. (2019). A conditionally immortalized Gli1-positive kidney mesenchymal cell line models myofibroblast transition. Am. J. Physiol. Renal. Physiol..

[82] Pang P., Shimo T., Takada H., Matsumoto K., Yoshioka N., Ibaragi S., Sasaki A. (2015). Expression pattern of sonic hedgehog signaling and calcitonin gene-related peptide in the socket healing process after tooth extraction. Biochem. Biophys. Res. Commun..

[83] Harada H., Kettunen P., Jung H.S., Mustonen T., Wang Y.A., Thesleff I. (1999). Localization of putative stem cells in dental epithelium and their association with Notch and FGF signaling. J. Cell Biol..

[84] Ishikawa Y., Nakatomi M., Ida-Yonemochi H., Ohshima H. (2017). Quiescent adult stem cells in murine teeth are regulated by Shh signaling. Cell Tissue Res..

[85] Seidel K., Ahn C.P., Lyons D., Nee A., Ting K., Brownell I., Cao T., Carano R.A.D., Curran T., Schober M. (2010). Hedgehog signaling regulates the generation of ameloblast progenitors in the continuously growing mouse incisor. Development.

[86] Yamazaki H., Tsuneto M., Yoshino M., Yamamura K., Hayashi S. (2007). Potential of dental mesenchymal Cells in developing teeth. Stem Cells.

[87] Zhao H., Feng J., Seidel K., Shi S., Chai Y. (2014). Secretion of Shh by a neurovascular bundle niche supports mesenchymal stem cell homeostasis in the adult mouse incisor. Cell Stem Cell.

[88] Shi C., Yuan Y., Guo Y., Jing J., Ho T.V., Han X., Li J., Feng J., Chai Y. (2019). BMP signaling in regulating mesenchymal stem cells in incisor homeostasis. J. Dent. Res..

[89] Brownell I., Guevara E., Bai C.B., Loomis C.A., Joyner A.L. (2011). Nerve-derived Sonic hedgehog defines a niche for hair follicle stem cells capable of becoming epidermal stem cells. Cell Stem Cell.

[90] Feng J., Jing J., Li J., Zhao H., Punj V., Zhang T., Xu J., Chai Y. (2017). BMP signaling orchestrates a transcriptional network to control the fate of mesenchymal stem cells in mice. Development.

[91] Li C., Jing Y., Wang K., Ren Y., Liu X., Wang X., Wang Z., Zhao H., Feng J.Q. (2018). Dentinal mineralization is not limited in the mineralization front but occurs along with the entire odontoblast process. Int. J. Biol. Sci..

[92] Mimeault M., Hauke R., Batra S.K. (2007). Stem cells: A revolution in therapeutics-recent advances in stem cell biology and their therapeutic applications in regenerative medicine and cancer therapies. Clin. Pharmacol. Ther..

[93] Picinich S.C., Mishra P.J., Mishra P.J., Glod J., Banerjee D. (2007). The therapeutic potential of mesenchymal stem cells. Cell- & tissue-based therapy. Expert. Opin. Biol. Ther..

[94] Hosoya A., Yoshiba K., Yoshiba N., Hoshi K., Iwaku M., Ozawa H. (2003). An immunohistochemical study on hard tissue formation in a subcutaneously transplanted rat molar. Histochem. Cell Biol..

[95] Hosoya A., Yukita A., Yoshiba K., Yoshiba N., Takahashi M., Nakamura H. (2012). Two distinct processes of bone-like tissue formation by dental pulp cells after tooth transplantation. J. Histochem. Cytochem..

[96] Hosoya A., Ninomiya T., Hiraga T., Zhao C., Yoshiba K., Nakamura H. (2008). Alveolar bone regeneration of subcutaneously transplanted rat molar. Bone.

[97] Hasan M.R., Takebe H., Shalehin N., Obara N., Saito T., Irie K. (2017). Effects of tooth storage media on periodontal ligament preservation. Dent. Traumatol..

